# Development of Resistance to Endoplasmic Reticulum Stress-Inducing Agents in Mouse Leukemic L1210 Cells

**DOI:** 10.3390/molecules25112517

**Published:** 2020-05-28

**Authors:** Martin Cagala, Lucia Pavlikova, Mario Seres, Karolina Kadlecikova, Albert Breier, Zdena Sulova

**Affiliations:** 1 Institute of Molecular Physiology and Genetics, Centre of Biosciences, Slovak Academy of Sciences, Dúbravská cesta 9, 84505 Bratislava, Slovakia; martin.cagala@savba.sk (M.C.); mario.seres@savba.sk (M.S.); 2 Institute of Biochemistry and Microbiology, Faculty of Chemical and Food Technology, Slovak University of Technology in Bratislava, Radlinského 9, 81237 Bratislava, Slovakia; karolina.kadlecik@gmail.com

**Keywords:** endoplasmic reticulum stress, multiple drug resistance, L1210 cells, tunicamycin, thapsigargin, bortezomib, MG-132, vincristine, proteasomal activity

## Abstract

Four new variants of L1210 cells resistant to endoplasmic reticulum (ER) stressors, tunicamycin (S_Tun_), thapsigargin (S_Thap_), bortezomib (S_Bor_), and MG-132 (S_MG-132_), were developed via an 18-month periodic cultivation in culture medium with a gradual increase in substance concentration. Multidrug resistance was generated for S_Tun_ (to tunicamycin, bortezomib and MG-132), S_Thap_ (to tunicamycin, thapsigargin and MG-132), S_Bor_ (to bortezomib and MG-132), and S_MG-132_ (to bortezomib and MG-132). These cells were compared to the original L1210 cells and another two variants, which expressed P-gp due to induction with vincristine or transfection with the gene encoding P-gp, in terms of the following properties: sensitivity to either vincristine or the ER stressors listed above, proliferative activity, expression of resistance markers and proteins involved in the ER stress response, and proteasome activity. The resistance of the new cell variants to ER stressors was accompanied by a decreased proliferation rate and increased proteasome activity. The most consistent change in protein expression was the elevation of GRP78/BiP at the mRNA and protein levels in all resistant variants of L1210 cells. In conclusion, the mechanisms of resistance to these stressors have certain common features, but there are also specific differences.

## 1. Introduction

Multidrug resistance (MDR) represents a real obstacle in the effective chemotherapy of leukemia patients; therefore, understanding the mechanisms of its development and finding molecular markers with sufficient predictive properties represent important goals [1,2]. The development of MDR in neoplastic cells involves several molecular features that may be present in the transformed cells from the beginning (intrinsic MDR) or may be developed in cells after exposure to cytotoxic agents (acquired MDR, reviewed in [3,4]). There are several well-defined molecular mechanisms that confer cells with loss of sensitivity to anticancer drugs, of which the following are prevalent: i. elevation of drug metabolism via increases in the activity/expression of oxidizing enzymes of the first phase and conjugating enzymes of the second phase of cell detoxification [5]; ii. elevation of cell drug efflux caused by upregulation of the expression/activity of plasma membrane efflux pumps, particularly members of the ABC (ATP-binding cassette) gene family [3,4]; iii. alterations of the initiation and progression of drug-induced programs of cell death [6]; iv. alteration in activity/expression of enzymes involved in DNA repair mechanisms [7]; and v. altered differentiation status caused by aberrant DNA methylation [8].

The most often observed molecular causes of MDR are overexpression of *P*-glycoprotein (ABCB1, a member of the ABC transporter gene family), a drug efflux pump in the plasma membrane that ensures the effective expulsion of P-gp substrates from the inner space of cells (reviewed in [3]). The *P*-glycoprotein is a 145 kDa polypeptide consisting of two similar tandemly linked halves. Each half contains a transmembrane domain consisting of six α-helical spans and an ATP binding and hydrolyzing domain with the ABC motif sequence. *P*-glycoprotein is *N*-glycosylated to 170–180 kDa on first extracellular loop. A characteristic feature of P-gp is the broad substrate specificity for various substances with basically different chemical structure [3]. Several lines of evidence have indicated that alteration of the cell response to endoplasmic reticulum (ER) stress, i.e., the accumulation of unfolded proteins within the ER, should change the sensitivity of cells to drugs, and this phenomenon may cooperate with the typical MDR phenotype when P-gp is overexpressed [4]. We recently found that L1210 cell variants overexpressing P-gp have elevated cell levels of GRP78/BiP, an ER stress regulator that is responsible for a depressed response to the ER stressor tunicamycin [9].

A later study utilized three variants of murine L1210 leukemia cells: a parental variant (S) negative for P-gp expression; an R variant in which P-gp expression was induced by selection with vincristine (VCR); and a T variant in which expression of P-gp was induced by transfection with the human ABCB1 gene [10]. P-gp-positive cell variants exhibited resistance to the following agents in addition to typical resistance to P-gp substrates: concanavalin A [10], thapsigargin (Thap) [9,11], cisplatin [12] and tunicamycin (Tun) [9,13,14]. However, resistance to these four substances was not related to the efflux of *P*-glycoprotein.

Two of the four substances (Thap and Tun) are known as inducers of ER stress, which they accomplish by either altering the Ca^2+^ concentration in the ER lumen, causing subsequent limitation of the functions of calnexin and calreticulin [15] or directly inhibiting the initial step of *N*-glycosylation in the ER [16]. Both of these agents, although activated by different causes, induce an increase in unfolded proteins in the ER, triggering a typical cellular response, to unfolded protein (UPR), that results in ER stress. One way the cell solves this situation is to accelerate the degradation of unfolded proteins via the proteasome under the endoplasmic reticulum-associated degradation (ERAD) pathway [17]. Therefore, the inhibition of proteasome activity will exacerbate (and induce) ER stress [18]. We focused on two proven proteasome inhibitors, bortezomib (Bor) and MG-132 [18].

Regarding the reduced response of cells to ER stressors, we hypothesize that there must exist specific mechanisms in cells capable of attenuating the cell response to the accumulation of unfolded proteins. Therefore, we prepared variants of S cells resistant to the ER stressors described above, in which the level of P-gp was not massively increased, and compared them with P-gp positive R and T cells. To achieve this goal, we studied the response of S cells to persistent stress caused by culturing at stepwise increasing concentrations of tunicamycin, thapsigargin, bortezomib and MG-132.

These experiments allow us to study mechanisms of resistance to ER stress induced by individual stressors. Although ER stress associated with these substances results in the accumulation of unfolded proteins in the ER and initiation of the UPR and the ERAD pathway, each of these stressors specifically induces ER stress [19]. As a result, four new variants of L1210 cells were obtained that are able to grow in high concentrations of one of the four substances mentioned above: thapsigargin (S_Thap_), tunicamycin (S_Tun_), bortezomib (S_Bor_) and MG-132 (S_MG-132_). The characteristics of these cell variants are described herein.

## 2. Results

### 2.1. Characterization of L1210 Cell Variants Resistant to Tun, Thap, Bor and MG-132 and their Comparison with S, R and T Cells.

S cells were exposed to ER stressors during repeated passages over one and a half years in culture medium with a gradually increasing concentration of one of the four stressors: Tun, Thap, Bor and MG-132. This process was started with initial concentrations of 10 nM Tun, 1 nM Thap, 1 nM Bor, and 10 nM MG-132. The final cell variants were able to proliferate in the immediately following passage in culture medium containing 10 µM Tun (S_Tun_ variant), 2 µM Thap (S_Thap_ variant), 0.1 µM Bor (S_Bor_ variant), and or 0.1 µM MG-132 (S_MG-132_ variant). The sensitivity of the original S cells to these substances and the prototypical P-gp substrate VCR are shown in Table 1. The biggest problems arose with cell adaptation to MG-132. Although S cells tolerated MG-132 at concentrations of about 100 nM (LC_50_ = 0.238 μΜ) when administered once, they did not tolerate repeated passages at these concentrations. During passage at much lower concentrations, which gradually increased, we were able to prepare cells capable of repeated passages in the presence of 0.1 μM MG-132. Under these conditions, S cells survive to a maximum of the third passage. We obtained S_MG-132_ cells with more than 10-fold higher LC_50_ value for MG-132 compared to S cells.

**Figure 1:**
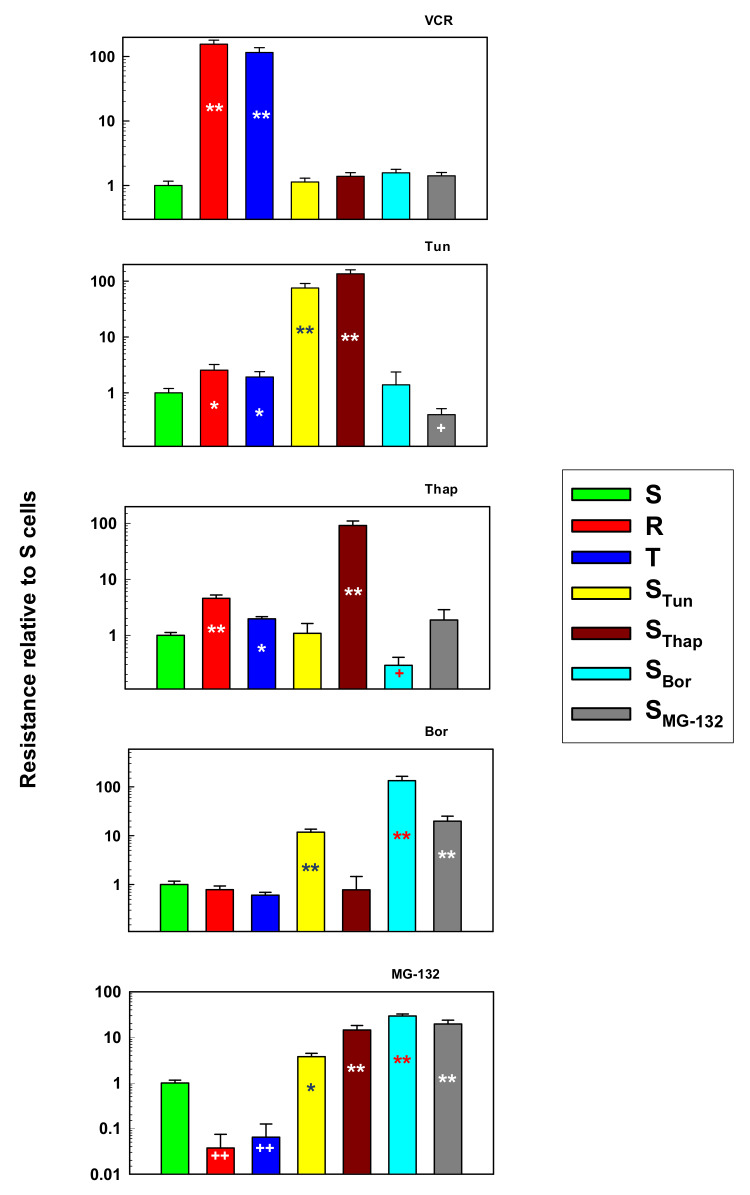
Sensitivities of S, R, T, S_Tun_, S_Thap_, S_Bor_ and S_MG-132_ cells to vincristine (VCR), tunicamycin (Tun), thapsigargin (Thap), bortezomib (Bor), and MG-132. Cells were cultivated for two days in the presence of these substances in a concentration range of 10^-4^–10 μM, and the LC_50_ values were calculated according to Equation (1) (See the Section 4). Data are expressed relative to the LC_50_ value for S cells, which was arbitrarily set as one. Cell survival was measured by the MTT test. Data represent the calculated value ± S_D_ for 27 degrees of freedom. Significance: * and ** significantly higher than the value obtained for S cells at *p* < 0.02 and *p* < 0.002, respectively. ^+^ and ^++^ significantly lower than the value obtained for S cells at *p* < 0.05 and *p* < 0.01, respectively.

Sensitivities to VCR, Tun, Thap, Bor and MG-132 of the cell variants obtained by the above described selection procedure were compared with those of S, R and T cells (Figure 1).

None of the newly prepared cell variants showed altered susceptibility to vincristine, to which *P*-glycoprotein-expressing R and T cells are strongly resistant. S_Tun_ and S_Thap_ cells are strongly resistant to tunicamycin, and mild resistance to this stressor is also observed in P-gp-positive R and T cells. In contrast, S_MG-132_ cells are more sensitive to Tun than their counterpart S cells. High Thap resistance was only seen in S_Thap_ cells, but less pronounced resistance was also found for P-gp-positive R and T cells. The three cell variants were resistant to bortezomib in the order S_Bor_ > S_MG-132_ > S_Tun_, and four cell variants were resistant to MG-132 in the order S_MG-132_ ≈ S_Bor_ > S_Thap_> S_Tun_. Both P-gp-positive R and T cells were more sensitive to MG-132 than the parental S cells.

**Figure 2:**
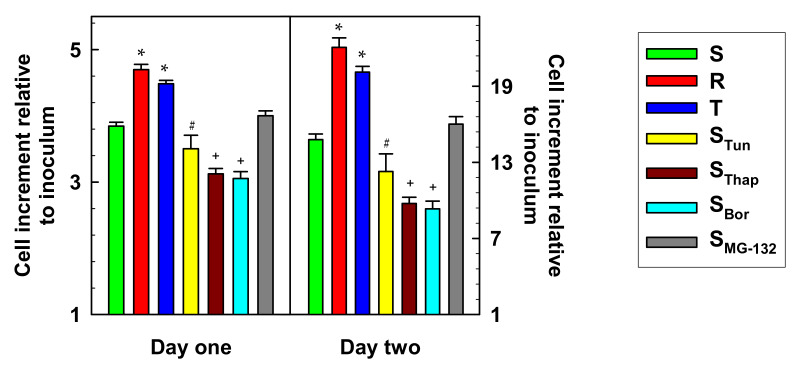
Cell growth after the first and second days of culture. All cell variants were cultured under standard conditions (see the Section 4). Data were calculated by nonlinear regression with Equation (2) according to the growth lines documented in Figure S1 (Supplementary files). Data represent the calculated value ± S_D_ for 28 degrees of freedom. Significance: * significantly higher than the value obtained for S cells at *p* < 0.02; ^#^ and ^+^ significantly lower than the value obtained for S cells at *p* < 0.05 and *p* < 0.02, respectively.

In further experiments, we measured the proliferation rate of all variants of L1210 cells. Over two days, the number of cells increased over time according to first-order kinetics (Equation (2), see the Section 4), thus giving a semilogarithmic plot providing a straight line for all L1210 cell variants (Figure S1 in Supplementary files). This result made it possible to determine the first-order rate constants shown in Figure S1 (Supplementary files). With Equation (2), we further calculated cell growth after the first and second days of culture, as shown in Figure 2.

The S_MG-132_ cell variant grew approximately as fast as the parental S cells (Figure 2). *P*-glycoprotein-positive R and T cell variants grew faster than parental S cells, and the S_Tun_, S_Thap_ and S_Bor_ variants grew more slowly than S cells.

### 2.2. Altered Expression of Genes Known to be Involved in Drug Metabolism/Elimination and in the ER Stress Pathway

**Figure 3:**
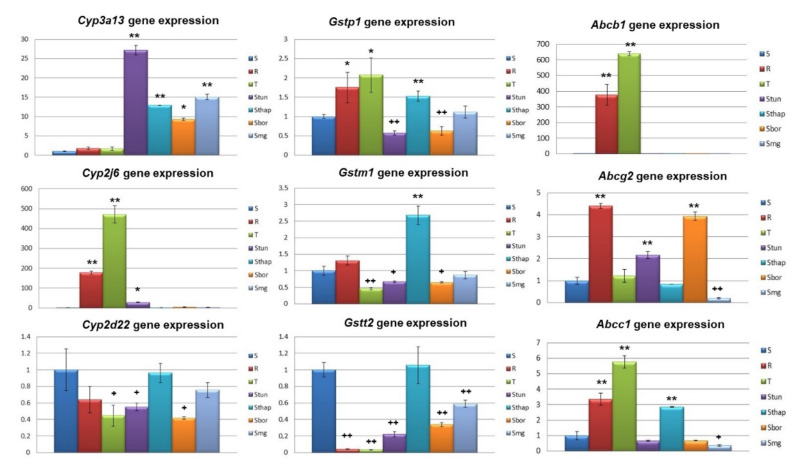
qRT-PCR quantification of *Cyp* (*Cyp3a13*, *Cyp2j6* and *Cyp2d22*), *Gst* (*Gstp1*, *Gstm1* and *Gstt2*) and *Abc* (*Abcb1*, *Abcg2* and *Abcc1*) gene expression (using the primers listed in Table 2 in S, R, T, S_Tun_, S_Thap_, S_Bor_ and S_MG-132_ cells. Transcript levels were normalized to the *β-actin* housekeeping gene and are expressed as the mean ± S_D_ of three independent measurements. Significance: Data are higher than those in S cells at * *p* < 0.02, ** *p* < 0.005; Data are lower than those in S at ^+^ *p* < 0.05, ^++^ *p* < 0.01.

There are three strictly regulated phases of cellular processing of harmful chemicals [20]. i. The first phase is oxidation, which is carried out by redox enzymes predominantly from the cytochrome P-450 family (Cyp); we focused on members of the Cyp2 subfamily (*Cyp2d22*, the ortholog of human *CYP2D6*, and *Cyp2j6*, the ortholog of human *CYP2J2*) and the Cyp3 subfamily (*Cyp3a13*, the mouse ortholog of human *CYP3A4*). ii. The second phase is conjugation, which is realized by conjugating enzymes, often glutathione S-transferases (GST); we focused on *Gstm1*, *Gstp1* and *Gstt2*. iii. The third phase is elimination, which is carried out by efflux pumps, most commonly, those from the ABC transporter family; we focused on P-gp (the *Abcb1* gene product), multidrug resistance associated protein 1 (MRP1, the *Abcc1* gene product) and breast cancer resistance protein (BCRP, the *Abcg2* gene product). The expression profiles of these genes detected by qRT-PCR are documented in Figure 3.

Increased expression of the Cyp3a13 gene (increased by 8–27 times) was detected in all new variants of L1210 cells in the order S_Tun_ > S_MG-132_ > S_Thap_ > S_Bor_. In contrast, such an increase in expression was not present in either of the P-gp-positive cell variants (R and T). Substantial overexpression of the *Cyp2j6* gene (increased by more than a hundred times) occurred in P-gp-positive R and T cells compared with parental S cells but was not present in the new cell variants. For *Cyp2d22*, compared with S cells, downregulation was observed in T, S_Tun_, S_Bor_ and R cells (a nonsignificant downregulation in R cells), while expression was not altered in S_Thap_ and S_MG-132_ cells compared to S cells.

Changes in *Gst* gene expression in resistant cell variants ranged from 0.03 to 2.50 times the values observed for S cells (Figure 3). *Gstpl* was overexpressed in both P-gp-positive variants (R and T) and S_Thap_ cells compared to S cells. In contrast, this gene was underexpressed in S_Tun_ and S_Bor_ cells compared to S cells, and its expression reached almost the same level as that observed in S cells in S_MG-132_ cells. Overexpression of the *Gstm1* gene was observed only in the S_Thap_ cell variant, and in the T, S_Tun_ and S_Bor_ cell variants, this gene was underexpressed (Figure 3). In the other two cell variants (R and S_MG-132_), the changes in expression of the *Gstm1* gene compared to that in S cells were not significant. The *Gstt2* gene was underexpressed in almost all resistant variants of L1210 cells except S_Thap_ cells, in which its expression reached levels similar to those in parental S cells.

The expression of the gene encoding P-gp (*Abcb1*) was several hundred-fold higher in P-gp-positive cells (R and T) than in S cells or all other resistant cell variants (Figure 3). This considerable overexpression of P-gp is typical of multiple resistant cell models [3]. In S cells, the expression of this protein was difficult to detect (Figure 3). As in S cells, P-gp was not overexpressed in other variants of L1210 cells (S_Tun_, S_Thap_, S_Bor_ and S_MG-132_). Other ABC transporters were not as considerably overexpressed in all variants of L1210 cells as P-gp in P-gp-positive cells. In R, S_Tun_ and S_Bor_ cells, greater expression of the *Abcg2* gene was detected than in S cells. In contrast, expression of the *Abcg2* gene in S_MG-132_ cells was considerably reduced. Higher expression of the *Abcc1* gene, as in S cells, was observed in the R, T and S_Thap_ cell variants, and decreased expression of this gene was detected in S_MG-132_ cells.

**Figure 4:**
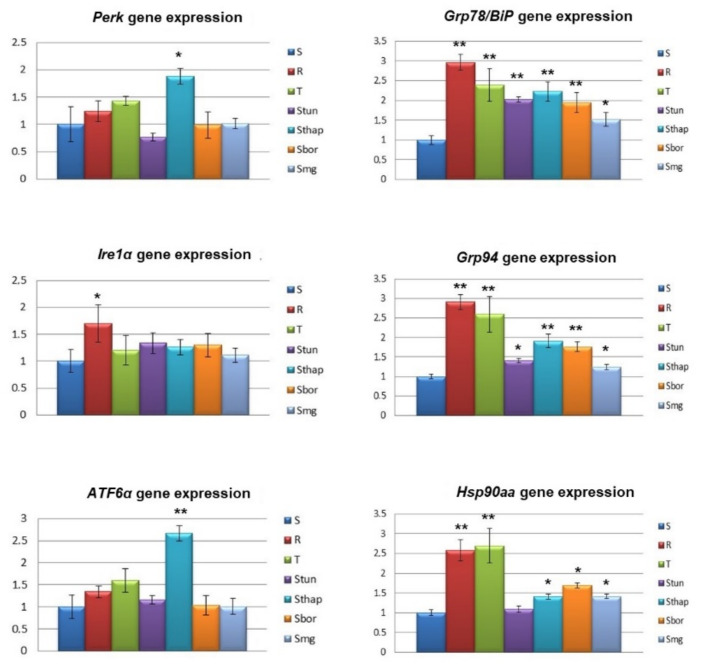
qRT-PCR quantification of protein kinase R (PKR)-like endoplasmic reticulum kinase (*Perk),* inositol-requiring enzyme 1α (*Ire1α)*, activating transcription factor 6α (*Atf6α)*, *Grp78/BiP*, *Grp94* and *Hsp90aa* gene expression (using primers listed in Table 2) in S, R, T, S_Tun_, S_Thap_, S_Bor_ and S_MG-132_ cells. Transcript levels were normalized to the *β-actin* housekeeping gene and are expressed as the mean ± S_D_ of three independent measurements. Significance: Data are higher than those in S cells at * *p* < 0.02, ** *p* < 0.005.

The cellular response to endoplasmic reticulum stress induced by the accumulation of unfolded proteins within the ER is mediated by three ER membrane receptors: protein kinase R (PKR)-like endoplasmic reticulum kinase (PERK), activating transcription factor 6α (ATF6α) and inositol-requiring enzyme 1α (IRE1α), whose activity is blocked by GRP78/BiP (glucose-regulated protein 78/binding immunoglobulin protein) under nonstress conditions [21]. During stress, GRP78/BiP dissociates from all three receptors, which are then activated and trigger subsequent processes. The response to ER stress is also regulated by the molecular chaperones GRP94 (glucose-regulated protein 94) and HSP90 (heat shock protein 90) [22]. Therefore, in further experiments, we studied the expression of these six proteins in all variants of L1210 cells. The expression levels of the three ER receptor genes (*Perk*, *Ire1α*, and *Atf6α*) in resistant L1210 cell variants were close to those obtained for S cells with predominantly nonsignificant differences (Figure 4). As exceptions, *Perk* and *Atf6α* were overexpressed in S_Thap_ cells, and *Ire1α* was overexpressed in R cells. *Grp78/BiP* expression was upregulated in all resistant variants of L1210 cells compared to parental S cells. Similarly, *Grp94* and *Hsp90aa* were overexpressed in all resistant variants of L1210 cells, except for S_Tun_ having nearly the same level of *Hsp90aa* than S cells.

### 2.3. Altered Cell Content of Proteins Known to be Involved in Drug Metabolism/Elimination and in the ER Stress Pathway

**Figure 5:**
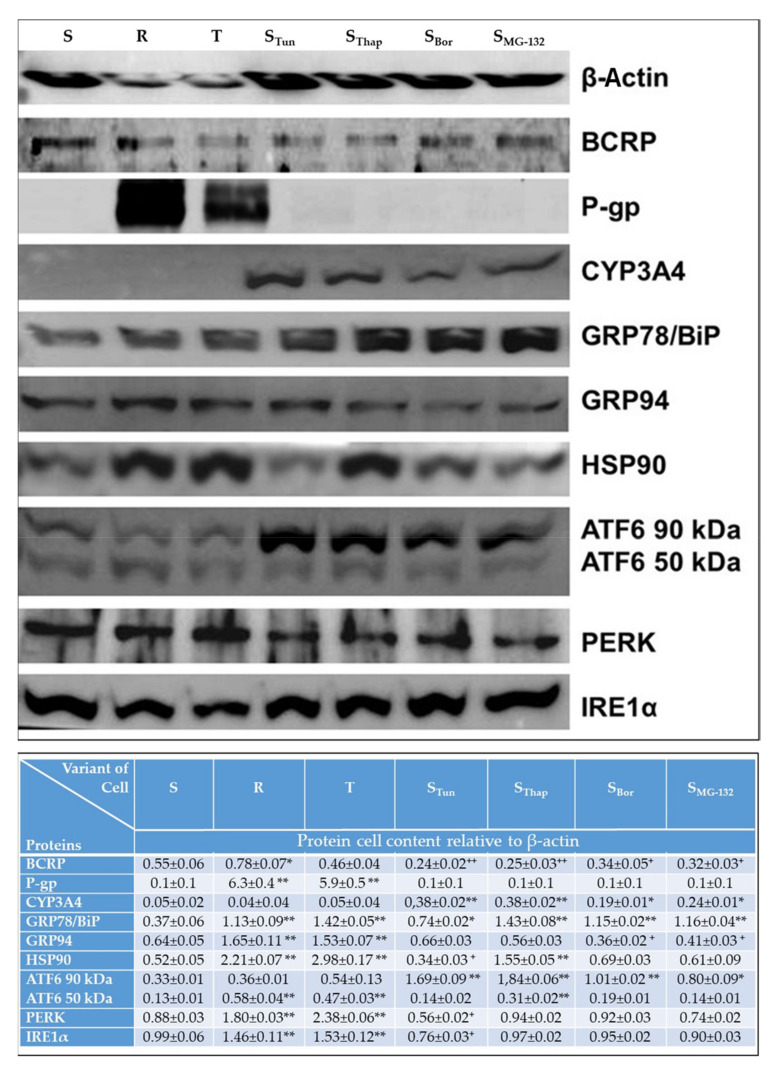
Western blot detection of cellular breast cancer resistance protein (BCRP), P-gp, CYP3A13 (detected by an antibody against its human ortholog, CYP3A4), GRP78/BiP, GRP94, HSP90, ATF6 (both the 90 and 50 kDa variants, PERK and IRE1α) levels. β-Actin was used as a control protein. Protein bands were quantified by densitometry, and data were normalized to β-actin cell content and are expressed as the mean ± S.E.M. of at least three independent measurements. Significance: values exceeded the corresponding value obtained for S cells at * *p* < 0.02, ** *p* < 0.005; values were less than the corresponding values obtained for S cells at ^+^ *p* < 0.05, ^++^ *p* < 0.02.

The cell levels of the selected proteins P-gp, BCRP, CYP3A13 (CYP3A4), GRP78/BiP, HSP90, ATF6 (full-length 90 kDa protein), ATF6 (50 kDa fragment), PERK and IRE1α were determined by Western blotting (Figure 5). We used an antibody against human CYP3A4 for the detection of CYP3A13, as CYP3A13 is the mouse ortholog of CYP3A4 with the highest (75%) degree of similarity (https://www.genecards.org/cgi-bin/carddisp.pl?gene=CYP3A4). Compared to the respective levels in S cells, significant upregulation was observed in the levels of P-gp in R and T cells, CYP3A13 in S_Tun_, S_Thap_, S_Bor_ and S_MG-132_ cells, and GRP78/BiP in all resistant cell variants. These results are consistent with the expression data of the corresponding genes documented in Figure 3. Furthermore, we detected an increase in the cell content of PERK and IRElα in R and T cells and, conversely, a decrease in these proteins in S_Tun_ cells. The level of ATF6 could not be accurately calculated because in addition to the full-length protein, we also observed its proteolytically truncated form, which is an active transcription factor that triggers the ER stress pathway [23]. Upregulation of GRP94 or HSP90 was observed in R and T cells or in R, T, and S_Thap_ cells, respectively (Figure 5). In contrast, the cellular levels of GRP94 were decreased in S_Bor_ or S_MG-132_ cells, as were the levels of HSP90 in S_Tun_ cells. The BCRP protein levels were near the value obtained for S cells. However, statistically significant differences were found for BCRP in some cell variants.

### 2.4. Proteasome Activity in L1210 Cell Variants

**Figure 6:**
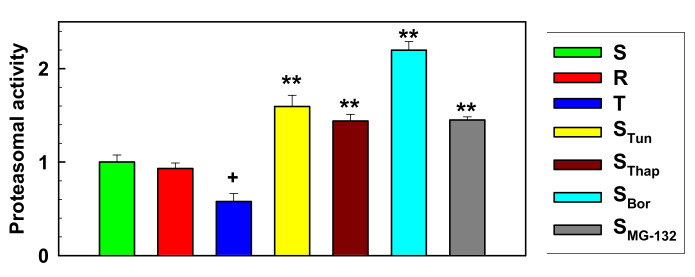
Proteasome activity as measured using the Proteasome Activity Assay Kit (ab107921). Data represent the computed value ± S_D_ according to the linear regressions in Figure S2 (in Supplementary files) for three independent measurements. Significance: Values are higher or lower than the corresponding values obtained for the S cell variant at ** *p* < 0.01 or ^+^ *p* < 0.05.

Proteasome activity was assessed fluorometrically with the Proteasome Activity Assay Kit (ab107921; Abcam, Cambridge, UK) in cell homogenates. This kit is based on the application of Suc-LLVY-AMC and MG-132 as a specific proteasomal substrate and inhibitor, respectively. The reaction was linear over 90 min of measurements (Figure S2 in Supplementary methods), which indicated a zero-order reaction typical of enzymatic reactions. This enabled the determination of the initial velocity of the reaction, which was constant during the full time of measurement, as the slope of the corresponding line. The values of the initial velocities of proteolytic activity of the proteasome as a measure of proteasomal activity are documented in Figure 6. Proteasome activity was upregulated in all newly prepared cell variants in the order S_Bor_ > S_Tun_ > S_Thap_ > S_MG-132_. In contrast, significant depression of proteasomal activity was registered for T cells compared with parental S cells, but for R cells, this value was not significantly different from that in parental S cells.

**Figure 7:**
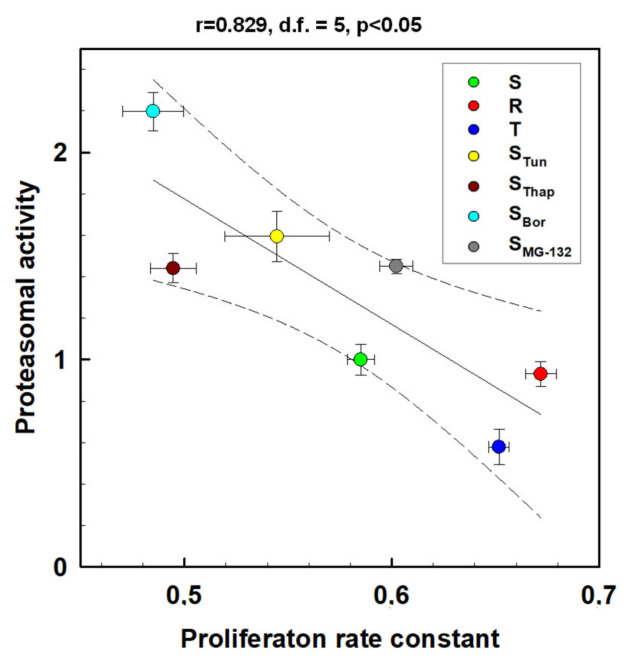
Correlation between proteasomal activity and proliferation rate constants of L1210 cell variants. The data used for the correlation analysis are documented in Figure 6 and Figure S1 (in Supplementary files) and represent value ± S_D._ The regression line (solid line) was characterized by a 0.95 confidence interval (dashed line). r-correlation coefficient, d.f.-degree of freedom.

Comparisons between the initial rates of proteasome proteolytic activity (Figure 6) and the cell proliferation rate constants (Figure S1 in Supplementary files) gave a statistically significant negative correlation (Figure 7, r = 0.829, *p* <0.05 for d.f. = 5). This suggests that in our L1210 cell variants, proteasome activity slowed the rate of cell proliferation by a yet unknown mechanism.

## 3. Discussion

**Figure 8:**
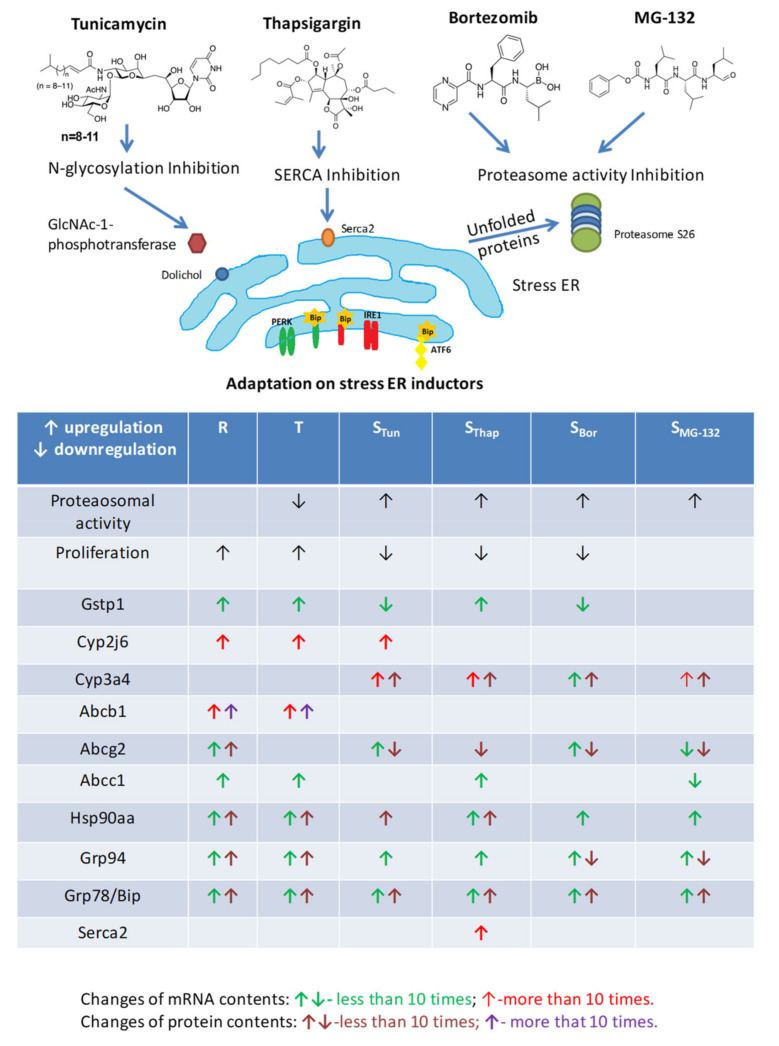
Chemical structures of tunicamycin, thapsigargin, bortezomib and MG-132 and their effects, which cause an increase in cell content of unfolded proteins. Structures were drawn using ACD/ChemSketch for academic and personal use (Advanced Chemistry Development, Inc. Toronto, Ontario, Canada). Tunicamycin is an inhibitor of GlcNAc-1-phosphotransferase (GPT), which catalyzes the transfer of *N*-acetylglucosamine-1-phosphate from UDP-*N*-acetylglucosamine to dolichol phosphate [24]. It is a mixture of homologous nucleoside antibiotics (they vary in the length of the side hydrocarbon chain) produced by several bacteria, including *Streptomyces clavuligerus* and *Streptomyces lysosuperificus*. Thapsigargin is a noncompetitive inhibitor of SERCA2-ATPase. It is a sesquiterpene lactone that naturally occurs in the plant *Thapsia garganica* [25]. Bortezomib is a high-affinity and specificity inhibitor of the 26S proteasome. It is an *N*-protected dipeptide, which stands for pyrazinoic acid, phenylalanine and Leucine with a boronic acid instead of a carboxylic acid and is a synthetic anticancer medication approved for the treatment of multiple myeloma and mantle cell lymphoma [26]. MG-132 is a potent, reversible, and cell-permeable 26S proteasome inhibitor [27]. It belongs to the class of synthetic peptide aldehydes. Induction of resistance by periodic passaging of S cells in media with stepwise increasing concentrations of Tun, Thap, Bor and MG132 provided resistant variants with altered expression of some proteins involved in the development of resistance and altered unfolded protein (UPR). The most important changes are summarized at the bottom of the figure.

In the present work, we describe novel variants of murine L1210 leukemia cells resulting from selection with ER stressors: the *N*-glycosylation inhibitor Tun, the ATPase inhibitor Thap, and the proteasome inhibitors Bor and MG-132. The chemical structure of these chemicals and their primary biological activities are documented in Figure 8.

Each of the variants obtained was able to proliferate at concentrations of the respective stressor to which it was accustomed of at least 10-fold higher than the concentration used with the original S line, and the process was completed over repeated passages. The properties of the novel cell variants were compared to those in parental cells, consisting of three variants of L1210 cells (S, R and T) suitable for studying the cytotoxic effects of substances in relation to P-gp expression [28]. In addition to significant resistance to VCR, which is a prototypical P-gp substrate, both P-gp-positive lines also showed moderate cross-resistance to Tun and Thap and, in contrast, hypersensitivity to MG-132 (Figure 1). The cross-resistance to Tun and Thap found in this study is consistent with the findings of our previous papers, in which the effect of Tun [9,13,14,29] and Thap [11,30,31] on S, R, and T cells was studied. This cross-resistance was also an incentive to carry out the experiments described in this work. None of the new cell variants showed cross-resistance to VCR (Figure 1) or overexpression of P-gp at the mRNA (Figure 3) or protein level (Figure 5).

S_Thap_ and S_Tun_ cells are strongly resistant to tunicamycin (Figure 1). However, S_Tun_ cells do not tolerate Thap and are as sensitive as S cells. This phenomenon can be explained as follows. Tunicamycin blocks *N*-glycosylation, which in turn blocks the processing of unfolded to properly folded proteins in the ER, thus inducing ER stress [32]. Thapsigargin primarily blocks SERCA-ATPase, resulting in a loss of Ca^2+^ within the ER, thereby eliminating calcium deposition in the ER [15]. However, Ca^2+^ is essential for the proper function of Ca^2+^-dependent lectins of the ER, calnexin and calreticulin, which play an essential role in the protein folding process [33]. Therefore, both agents induce the accumulation of unfolded proteins in the ER and trigger ER stress. In adapting to both Tun and Thap, the cells had to cope with accumulation of unfolded proteins in the ER. Therefore, it is not surprising that S_Thap_ cells can handle Tun considerably better than S cells. However, cells exposed to Thap suffer primarily from malfunction of intracellular Ca^2+^-homeostasis, which affects a wide variety of processes, including the process of correct protein folding. Thus, in addition to accumulating unfolded proteins in the ER, S_Thap_ cells must also manage changes in the proper functioning of intracellular calcium homeostasis, for which S_Tun_ cells have not developed adaptive mechanisms. Lee et al. [34] found that Thap-resistant PC3 cell variants derived from the original by incremental exposure to Thap had thirty-fold increased expression of the SERCA-2 gene. However, PC3 cells accustomed to either 10 nM or 2 µM Thap achieved similar levels of SERCA-2 overexpression, but they differed strongly in the level of resistance to Thap (60× and 1350×). Suppression of SERCA-2 expression with siRNA resulted in complete resistance reversal in cells accustomed to 10 nM Thap and only partial resistance reversal in cells accustomed to 2 µM Thap. Thus, overexpression of SERCA-2 was implicated in resistance to Thap but was not the only molecular cause of Thap resistance in PC3 cells with a high level of resistance [34]. Additionally, our S_Thap_ cells, as the only variant of L1210 cells studied, showed significant overexpression of the *Serca-2* gene (Figure S3, Supplementary files). S_Bor_ cells were strongly (over 100 times) resistant to Bor, and both S_MG-132_ and S_Tun_ cells were less (20-50 times) resistant to Bor. All new L1210 cell variants (S_Tun_, S_Thap_, S_Bor_ and S_MG-132_) were resistant to MG-132, but the P-gp-positive R and T cells showed significant hypersensitivity. While there are several literature-based explanations for these effects of Bor and MG-132, further targeted research will be needed to understand them accurately. Finally, it can be stated that the novel L1210 cell variants displayed resistance to multiple endoplasmic reticulum stressors: S_Tun_ (to Tun, Bor and MG-132), S_Thap_ (to Tun, Thap and MG-132), S_Bor_ (to Bor and MG-132), and S_MG-132_ (to Bor and MG-132).

All new variants of L1210 cells proliferated more slowly than S cells (Figure 2, Figure S1 in Supplementary files). In contrast, the P-gp-positive R and T cells showed faster growth than S cells. This could be related to changes in cyclin expression in our cell variants. In previous work, we detected enhanced expression of genes encoding cyclins D1 and E1 in R and T cells and only minor changes in the expression of cyclins A1 and B1 [9]. These results were obtained by classical RT-PCR methodology connected with identification of PCR products in agarose gel. Using more sensitive qRT-PCR methods, we detected significantly increased expression of cyclins B1, D1 and E1 in R cells and all four cyclins in T cells (Figure S4 Supplementary files). Of all the newly prepared cells, minor but significant overexpression of cyclin E1 was detected in only S_Thap_ cells. In all other cells (S_Tun_, S_Thap_, S_Bor_ and S_MG-132_), the expression of cyclins was either at a similar level or downregulated compared with the respective expression in S cells. While cyclin D1 is active in the transition from G1 to S phase of the cell cycle and its activity persists in all phases of the cell cycle [35], cyclin E1 is only active in the transition from G1 to S phase, [36], cyclin A is located in the nucleus during S phase and is involved in DNA replication [37], and cyclin B1 is G2/M-specific [38]. Therefore, according to these data, we note that R and T cells, which had a higher proliferative rate than S cells, had increased regulation of cyclin gene expression, and in S_Tun_, S_Thap_, S_Bor_ and S_MG-132_ cells, which proliferate more slowly than S cells, the gene expression of cyclins was downregulated.

In R and T cells, we observed massive expression of *P*-glycoprotein at both the mRNA and protein levels (Figure 3 and Figure 5), which is consistent with our previous work [10,12,14]. In other variants of L1210 cells, we did not observe the expression of this transporter. We also observed changes in *Abcc1* expression (overexpression in S, R and S_Thap_ cells; underexpression in S_MG-132_ cells) and *Abcg2* expression (overexpression in R, S_Tun_ and S_Bor_ cells; underexpression in S_MG-132_ cells). These changes may contribute to the overall resistance of our cells. However, the expression of both transporters did not exceed more than six times that obtained for S cells. When adapting Jurkat T-ALL cells to VCR, resistance associated with the overexpression of ABCC1 developed without enhancing ABCB1 expression [39]. In this case, the ABCC1 transporter was a prevalent cause of resistance, and its expression measured by an RNA expression chip was 30 times higher than that obtained for the parent line. Similarly, if the ABCG2 transporter is the main cause of resistance, its expression should be higher. As an example, the results of Volk et al. [40] have shown that ABCG2 gene expression levels in the range of 20-100-fold increase resistance at a multiple expression:multiple resistance ratio of either 1:1 for mitoxantrone or 10:1 for methotrexate.

Expression of the P-gp and CYP3A subfamily members often occurs under the transcriptional control of the xenobiotic nuclear receptors pregnane X receptor and constitutive androstane receptor. Therefore, it was surprising that *Cyp3a13* (a mouse ortholog of the human *CYP3A4* gene) overexpression was absent in R and T cells (Figure 3). Similarly, we did not observe enhancement of the immunoreactivity of the protein band labeled with an antibody against human CYP3A4 that shows cross-reactivity with murine CYP3A13 in R and T cells (Figure 5). In contrast, in new variants of L1210 cells (S_Tun_, S_Thap_, S_Bor_ and S_MG-132_), which did not express P-gp, we observed CYP3A13 overexpression at both the mRNA and protein levels (Figure 3 and Figure 5). The elevated CYP3A13 level may be partially responsible for the high cross-resistance of S_Tun_, S_Thap_ and S_Bor_ cells to MG-132 since this substance is rapidly metabolized by members of the CYP3A family [41]. In contrast to *Cyp3a13* gene expression, overexpression of the sixth member of the cytochrome P450 J-subfamily was found in the P-gp-positive R and T cells but was very low in other cell variants. *Abcb1* gene expression positively correlated with the expression of the *Cyp2j6* gene (Figure S5 Supplementary files), suggesting at least an indirect relationship between the expression of both genes. In humans, we know only one ortholog of the CYP2J subfamily—*CYP2J2* [42]. The product of this gene, in addition to its primary function in catalyzing the epoxygenase reaction, in which acceptors are polyunsaturated fatty acids (such as arachidonic acid), is able to metabolize various anticancer drugs and thus can influence processes important in overall cell resistance [43]. In humans, CYP2D6 is the only member of the cytochrome CYP2D subfamily. The number of drugs metabolized primarily by CYP2D6 is very large (~15–25% of all clinically used drugs), and they include different anticancer agents [44]. In mice, the situation is different: nine different Cyp2D family genes exist, and their silencing by appropriate siRNA has already been studied [45]. In our experiments, the *Cyp2d22* gene was never overexpressed in any variant of L1210 cells, and significant underexpression was detected in T, S_Tun_ and S_Bor_ cells. Therefore, this Cyp2D family member is unlikely to contribute to the resistance of L1210 cell variants.

In terms of GST family members, we detected overexpression of the *Gstp1* gene in R, T and S_Thap_ cells and the *Gstm1* gene in S_Thap_ cells. In contrast, underexpression was determined for the *Gstp1* gene in S_Tun_ and S_Bor_ cells; for the *Gstm1* gene in T, S_Tun_ and S_Bor_ cells; and for *Gstt2* in all resistant variants of L1210 cells except S_Thap_ cells (Figure 3). Interestingly, the expression of the *Gstp1* gene positively correlated with the expression of the *Abcc1* transporter gene (Figure S5 Supplementary files). It is known that the products of both genes cooperate in protecting cells against toxic substances, e.g., etoposide, in A375 human malignant melanoma cells [46]. Moreover, Peklak-Scott et al. [47] found that GSTP1 confers low-level resistance (1.4–1.7-fold) to cisplatin-induced cytotoxicity in MCF7 cells. In R and T cells, we detected upregulation of the *Gstp1* gene (Figure 3), and we previously described low-level resistance (approximately two-fold) to cisplatin [12]. However, in a paper by Peklak-Scott et al. [47], the expression of MRP1 failed to augment or potentiate GSTP1-mediated resistance.

Under normal nonstress conditions, regulatory pathways of all three membrane receptors in the ER (PERK, IRE1α and ATF6α) are silenced by specific blockade with GRP78/BiP [48]. Unfolded proteins within the ER are dramatically increased during ER stress and compete with ER membrane receptors in the binding of GRP78/BiP. This causes the gradual release of ER membrane receptors from GRP78/Bip blockade and allows them to trigger downstream processes: attenuation of protein synthesis and activation of proteasomal degradation of unfolded proteins as pro-survival stimuli [49]. If these processes do not eliminate the massive excess of unfolded protein within the ER, death stimuli will prevail. We detected overexpression of GRP78/BiP at both the mRNA and protein levels in all resistant variants of L1210 cells (Figure 4 and Figure 5). In a recent paper, we showed that overexpression of GRP78/BiP was responsible for the altered response of R and T cells compared to S cells to tunicamycin [9]. Therefore, we hypothesize that overexpression of GRP78/BiP in S_Bor_, S_Thap_, S_Bor_ and S_MG-132_ cells contributes to the overall resistance of these cells to ER stressors. Changes in the expression of other important players in the cellular response to ER stress (Figure 4 and Figure 5) could be involved in alleviating the toxic effects of individual ER stressors on S_Tun_, S_Thap_, S_Bor_ and S_MG-132_ cells.

In all new variants of L1210 cells, we detected increased proteasome activity compared to S, R and T cells (Figure 6). Thus, these novel cell variants have more GRP78/BiP to bind both unfolded protein and ER receptors and moreover are able to more rapidly degrade unfolded proteins due to elevated proteasomal activity (Figure 8) and thus better tolerate ER stress. Both, overexpression of GRP78/BiP and activation of proteasome are prosurvival stimuli [50]. 

## 4. Materials and Methods

### 4.1. Chemicals

ER-stressors: Tunicamycin (IUPAC name: (*E*)-*N*-[(*2S*,*3R*,*4R*,*5R*,*6R*)-2-[(*2R*,*3R*,*4R*,*5S*,*6R*)-3-acetamido-4,5-dihydroxy-6-(hydroxymethyl)oxan-2-yl]oxy-6-[2-[(*2R*,*3S*,*4R*,*5R*)-5-(2,4-dioxopyrimi-din-1-yl)-3,4-dihydroxyoxolan-2-yl]-2-hydroxyethyl]-4,5-dihydroxyoxan-3-yl]-5-methylhex-2-ena-mide); thapsigargin (IUPAC name: (*3S*,*3aR*,*4S*,*6S*,*6aR*,*7S*,*8S*,*9bS*)-6-(acetyloxy)-4-(butyryloxy)-3,3a-dihydroxy-3,6,9-trimethyl-8-{[(*2Z*)-2-methylbut-2-enoyl]oxy}-2-oxo-2,3,3a,4,5,6,6a,7,8,9b-decahydro-azuleno [4,5-b]furan-7-yl octanoate); bortezomib (IUPAC name: ([(*1R*)-3-methyl-1-[[(*2S*)-3-phenyl-2-(pyrazine-2-carbonylamino)propanoyl]amino]butyl]boronic acid; MG-132 (*N*-Benzyloxycarbonyl-l-leucyl-l-leucyl-l-leucinal, IUPAC name benzyl *N*-[(*2S*)-4-methyl-1-[[(*2S*)-4-methyl-1-[[(*2S*)-4-methyl-1-oxopentan-2-yl]amino]-1-oxopentan-2-yl]ami-no]-1-oxopentan-2-yl]carbamate); vincristine (IUPAC name methyl (*1R*,*9R*,*10S*,*11R*,*12R*,*19R*)-11-acetyloxy-12-ethyl-4-[(*13S*,*15S*,*17S*)-17-ethyl-17-hydroxy-13-methoxycarbonyl-1,11-diazatetracyclo [13.3.1.0^4,12^.0^5,10^]nonadeca-4(12),5,7,9-tetraen-13-yl]-8-formyl-10-hydroxy-5-methoxy-8,16-diaza-pentacyclo [10.6.1.0^1,9^.0^2,7^.01^6,19^]nonadeca-2,4,6,13-tetraene-10-carboxylate; sulfuric acid and other chemicals, unless otherwise stated in the text, were supplied by the Merck group via MERCK spol. s.r.o. (Bratislava, Slovak Republic).

### 4.2. Cell Culture and Cultivation Conditions

The murine lymphocytic leukemia cell line L1210 (ACC-123, S) was obtained from Leibniz-Institut DSMZ-Deutsche Sammlung von Mikroorganismen und Zellkulturen GmbH (Braunschweig, Germany) and is referred to hereafter as S. Drug-resistant variants were prepared from S cells: i. R cells (P-gp-positive MDR cells) via cultivation in cultivation medium with gradually increasing VCR concentration [10]; ii. T cells (P-gp-positive MDR cells) via stable transfection with Addgene plasmid 10,957 (pHaMDRwt) [10], a retrovirus encoding full-length P-gp cDNA [51]; iii. S_Tun_ cells (Tun-resistant cells) via cultivation in cultivation medium with gradually increasing Tun concentration; iv. S_Thap_ (Thap-resistant cells) via cultivation in cultivation medium with gradually increasing Thap concentration; v. SBor (Bor-resistant cells) via cultivation in cultivation medium with gradually increasing Bor concentration; and vi. S_MG-132_ (MG-132-resistant cells) via cultivation in cultivation medium with gradually increasing MG-132 concentration. This procedure was carried out for one and a half years of persistent repeated passages of the cells.

All seven variants of L1210 cells were cultivated in RPMI 1640 medium containing 8% bovine fetal serum and 20 μg/L gentamycin (both from Gibco, Langley, OK, USA) in a humidified atmosphere with 5% CO_2_ in air at 37 °C. Cells with acquired resistance to ER stressors were cultivated in the cultivation medium containing the corresponding agents at the following concentrations: S_Tun_—10 µM Tun, S_Thap_—2 µM Thap, S_Bor_—0.1 µM Bor, and S_MG-132_—0.1 µM MG-132.

### 4.3. Cell Viability Assay Using MTT

The cells (5 × 10^4^ cells/well) were cultured in the presence or absence of VCR, Tun, Thap, Bor and MG-132 (at a concentration range of 10^−4^–10 μM) added directly into 200 μL of cultivation medium in 96-well cell culture plates. After 48 h of cultivation, cell viability was assessed using the MTT assay [52], which was performed by adding MTT ([3-(4,5-dimethyldiazol-2-yl)-2,5-diphenyltetrazolium bromide]) to a final concentration of 0.25 mg/mL per well. The cells were then incubated with MTT for 2 h. Next, the plates were centrifuged for 15 min (5,000× *g*), and the sediments were dissolved with dimethyl sulfoxide. The absorbance at 540 nm was measured using a Universal Microplate Spectrophotometer mQuant (BioTek Instruments, Inc., Winooski, VT, USA). Dose-response curves were fitted according to an exponential decay (Equation (1)) by nonlinear regression using SigmaPlot graphing software (version 8.00, Systat Software GmbH, Erkrath, Germany). Statistical significance was analyzed using an unpaired Student’s t-test. Validation of Equation (1) was previously described [28]:

(1) $$N\;={\;N}_{O}\times e^{\left[\ln\left(0.5\right)\times \left(\frac{c}{{\mathrm{LC}}_{50}}\right)\right]}$$

where N represents the MTT signal in the presence of the respective agents at a concentration c; and N_0_ represents the MTT signal in the absence of any agents. LC_50_ is the median lethal concentration of an agent when N = 0.5 × N_0_.

### 4.4. Cell Proliferation Assay

Variants of L1210 cells (10^5^ cells per well) were plated/seeded in 6-well culture plates (time 0) and cultured in a humidified atmosphere with 5% CO_2_ in air at 37 °C. The number of viable cells was detected in a CASY Model TT Cell Counter (Roche Applied Sciences, Madison, WI, USA) in triplicate at different time intervals between 0 and 48 h. The time course of cell proliferation over this time interval followed first-order kinetics, i.e., according to Equation (2):

(2) $$N=I\times 10^{k\times t}$$

where N is the number of cells after cultivation for time t, I is the number of cells in inoculum and k is the first-order kinetic constant.

### 4.5. Real-time RT-PCR Conditions

Cells S_Tun_, S_Thap_, S_Bor_ and S_MG-132_ after culturing in the presence of Tun, Thap, Bor and MG-132 were used as inoculum (10^6^ cells in 5 mL of culture medium in Petri dishes) for drug-free passage. S, R and T cells were used under similar conditions, but cells growing in the absence of drugs were used as inoculum. Total mRNA was isolated from variants of L1210 cells using TRI reagent (Molecular Research Center, Inc. Cincinnati, OH, USA) according to the manufacturer’s instructions. Reverse transcription was performed using the RevertAid™ H Minus First Strand cDNA Synthesis Kit (Thermo Fisher Scientific, Bremen, Germany) according to the manufacturer’s protocol. Primers (Table 2) and cDNA samples were mixed with iTaq Universal SYBR Green Supermix (Bio-Rad Laboratories, Hercules, CA, USA) for qPCR. For the thermal cycle reactions, a CFX96 Real-Time System C1000 Touch Thermal Cycler (BioRad, Laboratories, Hercules, CA, USA) was used with the following conditions: 95 °C for 10 min and then 39 cycles at 95 °C for 15 s and at 59 °C for 30 s. The relative amount for each transcript was calculated by a standard curve of cycle thresholds for cDNA samples and normalized to the amount of β-actin. The polymerase chain reaction (PCR) was performed in triplicate for each sample, after which all experiments were repeated twice. The data were analyzed with Bio-Rad CFX96T software. Baseline levels for each gene were computed automatically. The results were quantified from Ct values according to the formula ΔΔCt = ΔCt sample—Δc housekeeping gene.

### 4.6. Western Blotting

The protein levels were semiquantitatively determined by Western blotting. Cells prepared similarly as described in previous chapter, were harvested and lysed with SoluLyse reagent containing a protease inhibitor cocktail (both from Sigma-Aldrich, St. Louis, MO, USA) and centrifuged at 12,000× *g* for 10 min. Protein lysates (30 μg per lane isolated from S, S_tun_, S_Thap_, S_Bor_ and S_MG-132_ cells and 15 μg isolated from R and T cells) were separated by SDS–PAGE on a Mini-Protean gel electrophoresis system (Bio-Rad, Philadelphia, PA, USA). We applied a reduced amount of proteins from R and T cells due to massive overexpression of P-gp, because at higher concentrations of proteins applied from other cell variants, due to overload with P-gp, its densitometric quantification was difficult. Proteins were transferred by electroblotting to a polyvinylidene fluoride membrane (GE Healthcare Europe GmbH, Vienna, Austria) and identified by using the following primary and secondary antibodies: rabbit polyclonal primary antibodies against BCRP, human CYP3A4 with cross-reactivity to mouse CYP3A13, PERK, IRE1A, ATF6A, GRP78/BIP, and GRP94 (all from Santa Cruz Biotechnology, Dallas, TX, USA).

Monoclonal primary antibodies from Abcam (Cambridge, UK) were used for detection of P-gp and β-actin. Goat anti-mouse and anti-rabbit immunoglobulins linked with horseradish peroxidase from Santa Cruz Biotechnology were used as secondary antibodies. The proteins were visualized with an enhanced chemiluminescence detection system (GE Healthcare Europe GmbH, Vienna, Austria) using an Amersham Imager 600 (GE Healthcare). Broad-range protein molecular weight markers (Thermo Fisher Scientific, Bremen, Germany) were used for molecular weight estimations. The intensity of the protein bands was quantified by densitometry using Image Amersham™ image analysis software (GE Healthcare Europe GmbH, Vienna, Austria). All samples were analyzed in triplicate, and the intensity levels were normalized to β-actin as a housekeeping protein. Significance was established using an unpaired Student’s t-test.

### 4.7. Proteasomal Activity Assay

The proteasomal activity assay was performed on 2 × 10^6^ cells per well. All variants of L1210 cells were cultivated for 24 h without drug in cultivation medium, and proteasome activity in samples was measured using an ab107921 kit (Abcam) according to the manufacturer’s protocol. The assay is based on a proteolysis substrate (Suc-LLVY-AMC) and a proteasome inhibitor (MG-132). Suc-LLVY-AMC is an AMC-tagged peptide (IUPAC name: 4-[[(*2S*)-1-[[(*2S*)-1-hydroxyphenyl)-1-[(4-methyl-2-oxochromen-7-yl) amino]-1-oxopropan-2-yl] amino]-3-methyl-1-oxobutan-2-yl] amino]-4 methyl-1-oxopentan-2-yl] amino]-4-methyl-1-oxopentan-2-yl] amino]-4-oxobutanoic acid), from which highly fluorescent AMC is liberated by chymotrypsin-like proteolysis. Specific suppression of proteasomal activity by MG-132 permits differentiation of proteasome activity from other protease activities that may be present in samples. Fluorescence was measured on a BioTek™ Synergy™ microplate reader (equipped with an Ex/Em = 350/440 nm filter, Thermo Fisher Scientific, Bremen, Germany). Experiments were performed in three independent experiments.

## 5. Conclusions

We prepared new variants of L1210 cells (Figure 1) resistant to TUN (S_Tun_ and S_Thap_), Thap (S_Thap_), Bor (S_Tun_, S_Bor_ and S_MG-132_) and MG-132 (S_Tun_, S_Thap_, S_Bor_ and S_MG-132_). Moreover, we confirmed milder resistance to Tun and Thap in R and T cells in addition to strong resistance against VCR, which is consistent with previous work [9,11,13,14,30]. However, these cells are hypersensitive to MG-132 (Figure 1). All new cell variants established in this work (S_Tun_, S_Thap_, S_Bor_ and S_MG-132_) are not resistant to vincristine. Resistance to MG-132 can be explained most simply because S_Tun_, S_Thap_, S_Bor_ and S_MG-132_ cells are resistant and show increased CYP3A13 expression, increased proteasome activity and slowed proliferation compared to S cells. In contrast, cells hypersensitive to MG-123 (R and T) did not have altered CYP3A13 expression. Moreover, proliferation velocity was increased and proteasomal activity was unchanged or decreased in this two cell variants. We assume that both the change in CYP3A13 (an enzyme that metabolizes MG-132) and the change in proteasomal activity (the activity that MG-132 inhibits) are directly related to the sensitivity of the cells to MG-132. However, we do not assume such a direct relationship for proliferation velocity and sensitivity to MG-132. In previous work, we showed that overexpression of GPP78/BiP in R and T cells is responsible for their resistance to Tun [9]. However, this resistance is only slight. S_Tun_ and S_Thap_ cells, which are much more resistant to tunicamycin than R and T cells (Figure 1), have a similar level of this protein expression as R and T cells. In contrast, S_Bor_ and S_MG-132_ cells are sensitive to Tun, despite overexpression of GRP78/BiP. This means that GRP78/BiP overexpression is only partially responsible for S_Tun_ and S_Thap_ cells resistance to Tun. Changes in the expression of other ER stress modulating proteins, (some of which are documented in Figure 4 and Figure 5), may contribute to this resistance, but further targeted research is needed to fully understand it.

High resistance to Thap was observed only in S_Thap_ cells and much less pronounced resistance in R and T cells. After Thap application, cells must be able to cope with the stress induced by accumulation of unfolded proteins, which may be mediated by increased GRP78/BiP levels (in all resistant cell variants) and increased proteasome activity in (S_Tun_, S_Thap_, S_Bor_ and S_MG-132,_ Figure 8). However, this is not sufficient because S_Tun_, S_Bor_ and S_Mg-132_ show these characteristics (Figure 8) and they are not resistant to Thap (Figure 1). However, Thap primarily suppresses SERCA2 ATPase activity, which causes a changes in cell Ca^2+^ homeostasis. In S_Thap_ cells, we observed an increase in SERCA 2 expression (Figure S3). In the past, we pointed out changes in calcium homeostasis in P-gp-expressing cells (reviewed in [31]). Thus, appropriate changes of intracellular Ca^2+^ homeostasis together with overexpression of GRP78/BiP will depress cell sensitivity to Thap.

S_Tun_, S_Bor_ and S_MG-132_, but not S_Thap_ cells are resistant to bortezomib (Figure 1). We do not have an explanation why S_Thap_ cells sensitivity to Bor persists and is depressed in all three other variants. Interestingly, S_Thap_ cells retain sensitivity to Bor and S_Bor_ cells are even hypersensitive to Thap. These facts will be studied in future research.

## Figures and Tables

**Table 1:** Median lethal concentrations of vincristine and endoplasmic reticulum (ER) stressors for S cells.

Substance	LC_50_ nM	S_D_ nM
Vincristine	1.0	0.2
Tunicamycin	440.0	120.7 ^1^
Thapsigargin	11.8	3.6
Bortezomib	6.1	1.5
MG-132	238.4	72.5

The degrees of freedom were 27; LC_50_ values were calculated by nonlinear regression according to Equation (1) (See the Section 4).

**Table 2:** Structure of primers.

Gene	Gene alias	Primers		bp
*Gstm1*		Forward Reverse	5ʹ-CCGTATGTTTGAGCCCAAGT-3ʹ 5ʹ-CTCCTAGTGAGTGCCCGTGT-3ʹ	186
*Gstp1*		Forward Revers	5ʹ-TGCCACCATACACCATTGTC-3ʹ 5ʹ-GGTGAGGTCTCCATCCTCAA-3	185
*Gstt2*		Forward Reverse	5ʹ-GTACCAGGTGGCAGACCACT-3ʹ 5ʹ-GTTGCAGAACCAGGACCATT-3	203
*Cyp2d22*		Forward Reverse	5ʹ-CAGTGTCCAGAGATGGCAGA-3ʹ 5ʹ-AGGACAGGTTGGTGATGAGG-3ʹ	175
*Cyp2j6*		Forward Reverse	5ʹ-GAAGGGTGCCCTTGTTGTTA-3ʹ 5ʹ-ACCAACAGAGTCCTGGGATG-3ʹ	151
*Cyp3a13*		Forward Reverse	5ʹ-CAAGAATCGTCCCCAAGAAA-3ʹ 5ʹ-GAAAGGTGCAGCACACAAAA-3ʹ	218
*Abcb1 Mouse*		Forward Reverse	5ʹ-TGGGAACTCTGGCTGCTATT-3ʹ 5ʹ-GGCGTACGTGGTCATTTCTT-3ʹ	179
*ABCB1 Human*		Forvard Reverse	5′-GCAATGGAGGAGCAAAGAAG-3’ 5´-CCAAAGTTCCCACCACCATA-3´	150
*Abcc1*		Forward Reverse	5ʹ-ACCAGCAACCCCGACTTTAC-3ʹ 5ʹ-TGGTTTTGTTGAGGTGTGTCA-3ʹ	151
*Abcg2*		Forward Reverse	5ʹ-CCACGTGTTAGTACCAATGTCG-3ʹ 5ʹ-TTTCCGGACTAGAAACCCACT-3ʹ	151
*Ccna1*	*CycA1*	Forward Reverse	5ʹ-ACACAGACCCAAGGCTCACT-3ʹ 5ʹ-ACAGGGTCTCTGTGCGAAGT-3ʹ	122
*Ccnb1*	*CycB1*	Forward Reverse	5ʹ-GGTGACTTCGCCTTTGTGAC-3ʹ 5ʹ-CTACGGAGGAAGTGCAGAGG-3ʹ	125
*Ccnd1*	*CycD1*	Forward Reverse	5ʹ-AGCAGAAGTGCGAAGAGGAG-3ʹ 5ʹ-CAAGGGAATGGTCTCCTTCA-3ʹ	149
*Ccne1*	*CycE1*	Forward Reverse	5ʹ-GGAAAATCAGACCACCCAGA-3ʹ 5ʹ-AGGATGACGCTGCAGAAAGT-3ʹ	131
*Actb*	*β-actin*	Forward Reverse	5ʹ-TCGCCATGGATGACGATA-3ʹ 5ʹ-CACGATGGAGGGGAATACAG-3ʹ	110
*Atf6*	*Atf6α*	Forward Reverse	5ʹ-GAGCCGCACAGCTACCTAAC-3ʹ 5ʹ-CCCATACTTCTGGTGGCACT-3ʹ	121
*Eif2ak3*	*Perk*	Forward Reverse	5ʹ-CTGCTGCTTCTGTTCCTGCT-3ʹ 5ʹ-CCCCTAAGCCAAACACTGTC-3ʹ	106
*Ern1*	*Ire1α*	Forward Reverse	5ʹ-TGCATGCTGTTAGCAAGAGG-3ʹ 5ʹ-GACTGCCATCATTGGGATCT-3ʹ	120
*Hspa5*	Grp78/BiP	Forward Reverse	5ʹ-TTTTCTGATGTATCCTCTTCACCAGT-3 5ʹ-TTCAGCCAATTATCAGCAAACTCT-3ʹ	73
*Hsp90aa1*	*Hsp90aa*	Forward Reverse	5ʹ-GGG AGC TCA TCT CCA ATT CA-3ʹ 5ʹ-ATTGATGTGCAGCTCCTTCC-3ʹ	101
*Hsp90b1*	*Grp94*	Forward Reverse	5ʹ-GGGGAGGTCACCTTCAAGTC-3ʹ 5ʹ-TGAGGGGGAGATCATCGGAA-3ʹ	199

## Supplementary Materials

The following are available online. Figure S1: Time course of L1210 cell variant proliferation; Figure S2: Time course of proteasomal activity in variants of L1210 cells; Figure S3: Expression of Serca-2 and Serca-3 genes in variants of L1210 cells; Figure S4: Expression of cyclins A1, B1, D1 and E1 in variants of L1210 cells; Figure S5: Correlation between Abcb1 and Cyp2j6 or Abcc1 and *Gstp1* gene expression.

## Abbreviations

ABC	ATP-binding cassette
ABCB1	1st member of ABCB gene subfamily
ABCC1	1st member of ABCC gene subfamily
ABCG2	2nd member of ABCG gene subfamily
ATF6α	activating transcription factor 6α
BCRP	breast cancer resistance protein
Bor	Bortezomib
CYP	cytochrome P-450
ER	endoplasmic reticulum
ERAD	endoplasmic reticulum-associated degradation
GRP78/BiP	glucose-regulated protein 78/binding immunoglobulin protein
GRP94	glucose-regulated protein 94
GST	glutathione S-transferase
HSP90	heat shock protein 90
MDR	multidrug resistance
MG-132	*N*-Benzyloxycarbonyl-l-leucyl-l-leucyl-l-leucinal
MRP	multidrug resistance associated protein
MTT	[3-(4,5-dimethyldiazol-2-yl)-2,5-diphenyltetrazolium bromide]
PERK	protein kinase R (PKR)-like endoplasmic reticulum kinase
P-gp	*P*-glycoprotein
R	P-gp-positive L1210 cells induced for resistance by vincristine
S	P-gp negative drug sensitive parental L1210 cells
S_Bor_	P-gp negative L1210 cells induced for resistance by bortezomib
S_MG-132_	P-gp negative L1210 cells induced for resistance by MG-132
S_Thap_	P-gp negative L1210 cells induced for resistance by thapsigargin
S_Tun_	P-gp negative L1210 cells induced for resistance by tunicamycin
Suc-LLVY-AMC	4-[[(*2S*)-1-[[(*2S*)-1-hydroxyphenyl)-1-[(4-methyl-2-oxochromen-7-yl) amino]-1-oxopropan-2-yl] amino]-3-methyl-1-oxobutan-2-yl] amino]-4 methyl-1-oxopentan-2-yl] amino]-4-methyl-1-oxopentan-2-yl] amino]-4-oxobutanoic acid)
T	drug-resistant P-gp-positive L1210 cells obtained by transfection with the human P-gp gene
Thap	Thapsigargin
Tun	Tunicamycin
UPR	unfolded protein response
VCR	Vincristine
