# Supplementary material for: Development of Resistance to Endoplasmic Reticulum Stress-Inducing Agents in Mouse Leukemic L1210 Cells
Source: Molecules. 2020 May 28;25(11):2517. doi: 10.3390/molecules25112517 (PMC7321222; doi:10.3390/molecules25112517)
Supplement: Supplementary file 1 [file molecules-25-02517-s001.pdf]

Supplementary files.

Development of resistance to endoplasmic reticulum stress-inducing agents in mouse leukemic L1210 cells.

Martin Cagala [1], Lucia Pavlikova [1], Mario Seres [1], Karolina Kadlecikova [2], Albert Breier [1,2,*], and Zdena Sulova [1,*]

[1] Institute of Molecular Physiology and Genetics, Centre of Biosciences, Slovak Academy of Sciences, Dúbravská cesta 9, 84505 Bratislava, Slovakia
[2] Institute of Biochemistry and Microbiology, Faculty of Chemical and Food Technology, Slovak University of Technology in Bratislava, Radlinského 9, 81237 Bratislava, Slovakia

S1. Time course of L1210 cell variant proliferation.

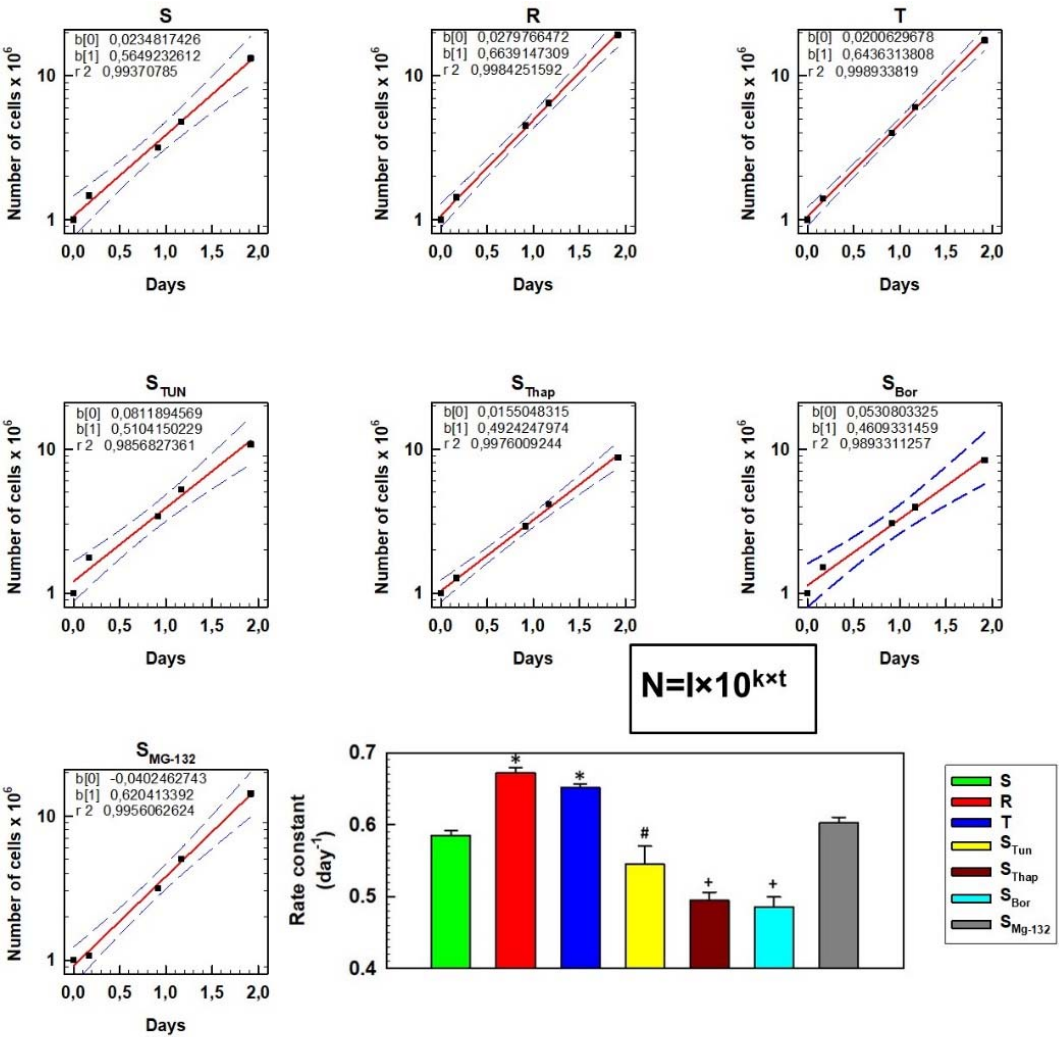

Figure S1. Kinetics of L1210 cell variant proliferation. The time courses of proliferation can be described by first-order kinetics for each cell variant and therefore gave a straight line in the semilogarithmic plot. Each point represents the average of 6 independent values, which were all used for correlation. Red solid line: regression line; blue dashed line: 95% confidence interval valid for 28 degrees of freedom. First-order rate constants (documented on the bar graph) were obtained by

nonlinear regression using the attached equation, where N is the number of cells at time T, and I is the number of cells in the inoculum. The rate constants are expressed as the calculated value±SD.

S2. Time course of proteasomal activity in variants of L1210 cells.

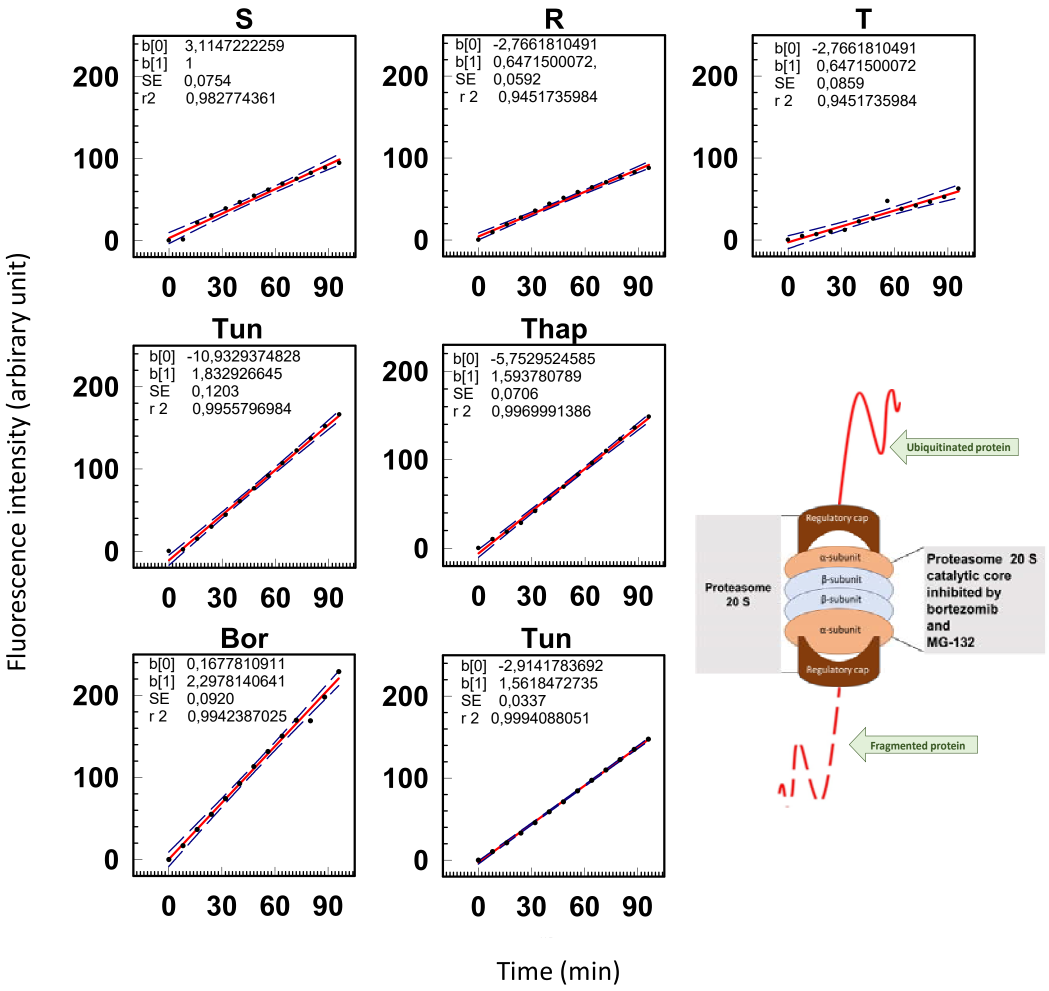

Figure S2. Time course of Suc-LLVY-AMC cleavage by the proteasome. The time courses can be described by zero-order kinetics, which is typical for enzyme reactions, for each cell variant and therefore gave straight lines in the graphs. The graphs are representative of three independent measurements. Red solid line: regression line; blue dashed line: 95% confidence interval valid for 11 degrees of freedom. The initial velocities of Suc-LLVY-AMC cleavage by the proteasome were obtained as the slope of the respective time course. The function of the proteasome and site where its inhibitors act are shown in the attached scheme.

S3. Expression of Serca-2 and Serca-3 genes in variants of L1210 cells

S3.1. RT-PCR detection of Serca2 and Serca3 expression

Isolation of total RNA and reverse transcription were performed with the same protocol as described in chapter 4.5.

PCR was performed in a 25 μl total volume containing 12.5 μl of 2x Taq green MasterMix, 10,5 μl of DNase-free water, 0,5 μl of 5 μM forward primer and 0,5 μl of 5 μM reverse primer. After treating the samples

at 94°C for 3 min to inactivate the reverse transcriptase, the samples were subjected to 30 cycles of 95°C for 30 s followed by 57 - 60°C for 30 s, depending on the primer used, and an incubation at 72°C for 90 s, and a final extension at 72°C for 10 min. The PCR products were separated in a 1.5% agarose gel (Lonza Group Ltd, Basel Switzerland) and visualized using GelRedTM nucleic acid gel stain (Thermo Scientific). Stained gels were imaged using an Amersham™ Imager 600 (GE Healthcare Bio-Science), and gel band quantification was processed by densitometry using Image Amersham™ software (GE Healthcare). The data were normalized to β-actin mRNA and are expressed as the mean±SD of three independent measurements.

Structure of primers.

Gene	Gene alias		Primers	bp
Atp2a2	*Serca2*	Forward	5'-CCAATGACAATGGCACTTTCT-3'	395
		Reverse	5'-GGGCTGGTAGATGTGTTGCT-3'	
Atp2a3	*Serca3*	Forward	5'-CGTATGGCACGTAAGAATGC-3'	190
		Reverse	5'-TGGTACCCGAAATGGTGAAT-3'	
Actb	β-actin	Forward	5'-TCGCCATGGATGACGATA-3'	110
		Reverse	5'-CACGATGGAGGGGAATACAG-3'	

S3.1. Serca2 and Serca3 expression

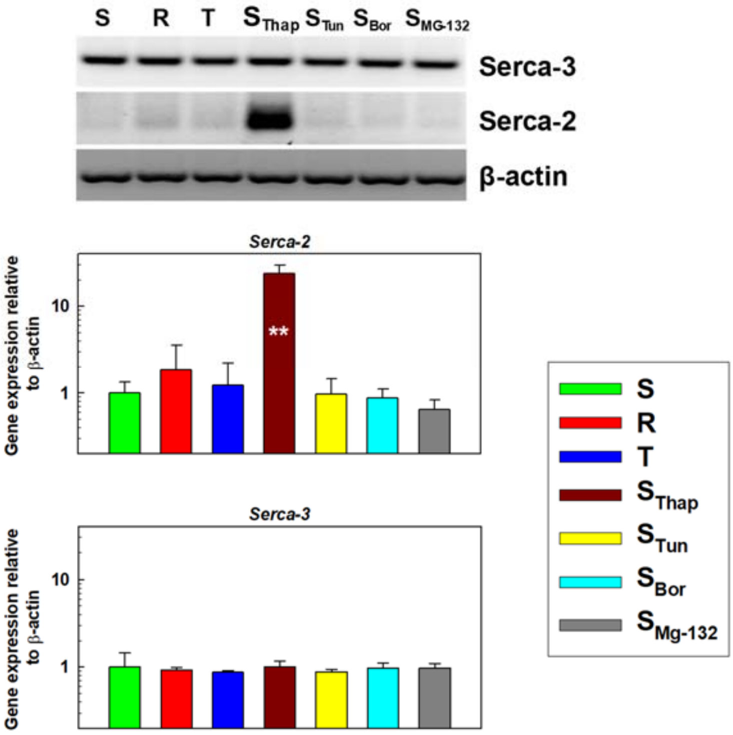

Figure S3. RT-PCR detection of *Serca2* and *Serca3* transcripts in variants of L1210 cells. *β-Actin* was used as an internal control. Data are representative of three independent measurements. Quantities of PCR products obtained by densitometry are expressed as the mean ± S.E.M. of three independent measurements of data relative to the *β-actin* signal. Only the value for *Serca2* in S_{Thap} cells significantly differed from that in S cells ($p<0.001$).

S4. Expression of cyclins A1, B1, D1 and E1 in variants of L1210 cells

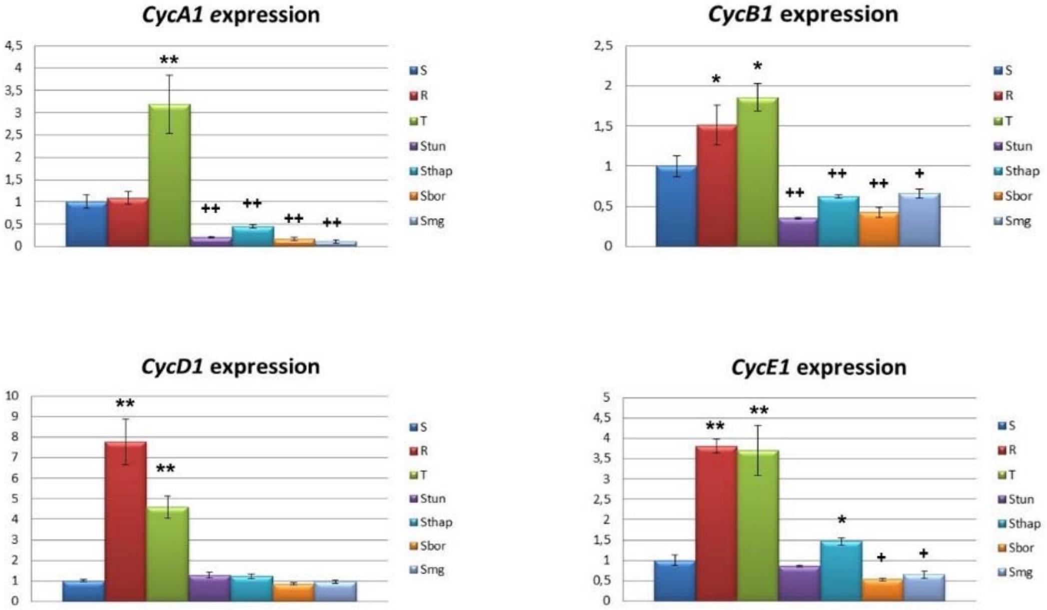

Figure S4. qRT-PCR quantification of *CycA1*, *CycB1*, *CycD1* and *CycE1* gene expression (using the primers listed in Table 1) in S, R, T, S_{Tun}, S_{Thap}, S_{Bor} and S_{MG-132} cells. Transcript levels were normalized to the *β-actin* housekeeping gene and are expressed as the mean ± SD of three independent measurements. Significance: Data are higher than those in S cells at * p<0.02, ** p<0.005; Data are lower than those in S cells at $^{+}$ p<0.05, $^{++}$ p<0.01.

S5. Correlation between *Abcb1* and *Cyp2j6* or *Abcc1* and *Gstp1* gene expression.

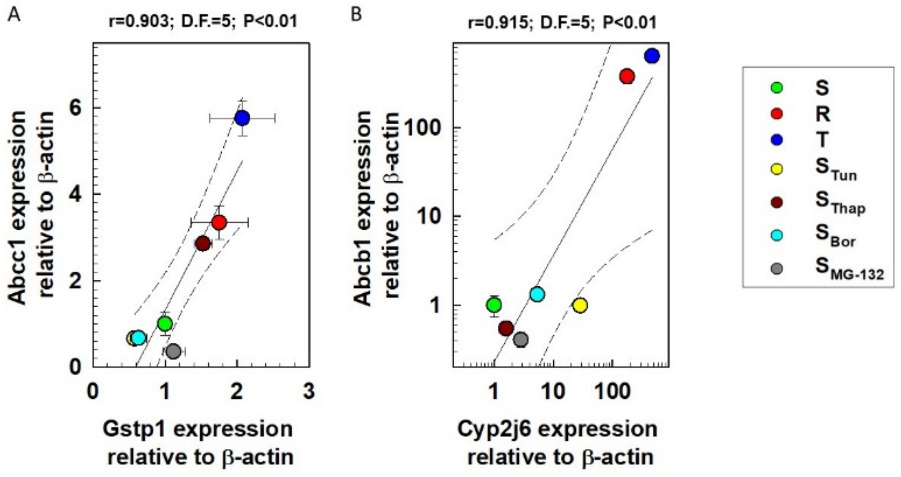

Figure S5. Correlation of *Abcc1* and *Gstp1* or *Abcb1* and *Cyp2j6* gene expression. Solid line: regression line; dashed line: 99% confidence interval.
